# Sports injuries in elite football players: classification, prevention, and treatment strategies update

**DOI:** 10.3389/fspor.2025.1643789

**Published:** 2025-09-08

**Authors:** Yinping Zeng, Weiguo Ji, Yu Shi, Wenduo Liu, Weiping Ji

**Affiliations:** ^1^ Department of Physical Education and Military Training, Zhejiang Ocean University, Zhoushan, China; ^2^ Department of Sports Science, College of Natural Science, Jeonbuk National University, Jeonju, Republic of Korea; ^3^ Department of Physical Education, Kunsan National University, Gunsan, Republic of Korea; ^4^ Faculty of Physical Education, Tomsk State University, Tomsk, Russia

**Keywords:** hamstring injury, anterior cruciate ligament (ACL) injury, tendon injury, joint sprain, muscle imbalance, degenerative injuries, concussion, fracture

## Abstract

Elite football players are particularly vulnerable to sports injuries due to the demands of high-intensity training and competition, which negatively affect their athletic careers and the economic interests of their clubs. Currently, the structural classification of injury scenarios and types in football remains unclear, hindering players' understanding of injuries and the implementation of effective preventive measures. This study aims to refine the structural classification of football-related injuries and update the corresponding scenarios, prevention strategies, and treatment approaches for general sports injuries, degenerative injuries, and accidental injuries. Researchers screened relevant literature from PubMed, SportDiscus, and Google Scholar databases from May 2000 to May 2025. For general sports injuries, skeletal muscle injuries (muscle fiber injuries, tendon injuries) can be prevented through eccentric strength training, while joint injuries (ligament injuries, muscle imbalances) require a focus on neuromuscular control training. Degenerative injuries necessitate systematic treatment, and surgical intervention should be adopted, when necessary, followed by a personalized rehabilitation program. Accidental injuries (concussions and fractures) can be mitigated using protective gear, rule modifications, and enhanced safety measures education. This article emphasizes the importance of a structured classification system for injury prevention and differentiated treatment strategies for elite football players. This will provide a theoretical basis for establishing clear understanding among individual athletes and reducing the occupational risk of injury in football.

## 1 Introduction

According to official Fédération Internationale de Football Association (FIFA) data, football is one of the most popular sports in the world, with the 2022 World Cup alone attracting a global audience of 5 billion viewers across various media platforms (1). Currently, the total number of professional athletes registered with FIFA is 128,876 (2). This study primarily focuses on elite-level football players. In the field of sports science, the definition of elite athletes has long been a subject of debate due to the diversity of sports and the complexity of evaluation criteria. Swanna et al. (2015), proposed a five-dimensional classification scoring system (competitive standards, competitive success, experience, the competitiveness of the athlete's home country, and the global competitiveness of the sport) to define the scope of elite athletes (3). William et al. (2017), advocate prioritizing quantitative performance data (such as personal bests) and only referencing competitive experience and success in high-level leagues when such data is unavailable (e.g., in team sports), rather than international representation (4). Zentgraf et al. (2024), analyzed and compared elite and semi-elite athletes based on neurophysiological indicators, characterizing elite athletes as “high-performance outliers” within their specific sports, emphasizing their specialization and international level (5).

Based on the aforementioned studies, we define elite athletes as individuals who have reached national or international competitive levels in their specific sport, consistently participate in high-level competitions, and possess a long-term background of organized, specialized training. They are generally engaged in professional leagues, national teams, or the Olympic framework, exhibiting exceptional competitive prowess and a robust will to succeed, and are considered “high-performance outliers” in their domain. This elite group, owing to extended exposure to intense competition and training loads, encounters markedly elevated injury risks compared to average athletes (6), rendering the occurrence and prevention of their sports injuries a matter of considerable concern.

To avoid conceptual overlap with the “contact/non-contact” mechanism-based classification, this study adopts a structural framework of “General Sports Injuries—Degenerative Injuries—Accidental Injuries,” in order to better serve practical decision-making in injury prevention and rehabilitation. Among these, “General Sports Injuries” primarily refer to those that can be reduced in incidence or severity through planned physical and technical training, and are predominantly non-contact in mechanism (e.g., muscle, tendon, and ligament injuries) (7). “Degenerative Injuries” are also mostly derived from non-contact mechanisms (such as tendinopathy, degenerative meniscus injury, etc.) (8). It is worth noting that in elite athletes, acute traumatic meniscus tears are also frequently observed. Although such injuries are usually categorized as contact injuries, they deserve particular attention because they involve not only preventive measures but also post-treatment rehabilitation (9). Regarding contact injuries, due to their high degree of randomness and difficulty in preventing them through training, this paper categorizes them under “Accidental Injuries”.

General sports injuries in football include muscle/tendon injury, joint (non-bone)/ligament injury, contusion, fracture, and laceration (10). Among these, nearly one-third are muscle injuries, and the majority (92%) affect the lower extremity (11). According to previous studies, players' performance in high-intensity exercise and technical skills usually significantly declines when they suffer moderate to severe muscular injuries (12). Therefore, current research primarily focuses on muscle and ligament injuries in the lower limbs and their prevention. Muscle injuries vary in their mechanisms, and prevention strategies also differ. Thus, this study divides muscle injuries into tendon injuries and muscle fiber injuries based on their anatomical characteristics. Hamstring strains, for example, primarily occur during high-speed movements or excessive stretching (13), with eccentric strengthening protocols demonstrating preventive efficacy (14). Additionally, tendon injuries often occur during high-impact activities such as sprinting and jumping (14), with some studies indicating that balance training may reduce the risk of tendon injuries (15). Furthermore, anterior cruciate ligament (ACL) tears have been extensively studied due to their severity and prolonged rehabilitation. These injuries can occur during deceleration and acceleration movements, primarily due to excessive loading of the ACL (16). Neuromuscular training has been widely employed in the prevention of ACL injuries, with previous research indicating its positive effects on improving lower limb muscle control and reducing injury risk (17). In summary, current evidence suggests that certain muscle injuries (muscle fiber and tendon) and ligament injuries in football can be effectively prevented through scientifically designed exercise interventions.

Football-related injuries include more complicated pathological disorders, including tendinopathies and articular cartilage lesions in addition to common sports injuries (8). Long recovery times and complicated aetiologias are common characteristics of these degenerative injuries, which frequently require medication or surgery. For instance, meniscal lesions are among the most common degenerative injuries in football players (18), with their pathogenesis typically ascribed to the combination of compressive force and transverse-plane tibiofemoral rotation as the knee transitions from flexion to extension during rapid cutting or pivoting (19). Depending on the type and severity of the meniscal injury, treatment options may include meniscectomy, meniscus repair, or meniscal reconstruction (19). To safely return to play, postoperative patients need systematic rehabilitation programs that gradually improve lower limb stability and joint function.

In addition to the aforementioned preventable injuries occurring during training and competition, football also involves accidental injuries, such as muscle cramps, concussions, and fractures (10). The occurrence of muscle cramps may be related to disturbances in hydration and electrolyte balance or abnormal spinal reflex activity caused by sustained muscle fatigue; however, the precise mechanisms remain unclear (20). According to earlier research, taking supplements containing trace elements (calcium, potassium, sodium) and drinking enough water may help reduce muscle cramping (21). Increased safety awareness during games and better instruction on safety procedures may help minimize accidental injuries (such as fractures and concussions).

In elite football, the occurrence of injury events can result in substantial medical costs, negatively impact team performance, and even prematurely end an athlete's professional career (22). Consequently, reducing football-related injuries is essential to protecting players' health and clubs’ financial stability (22). By categorizing the most frequent injuries in professional football into three main categories: general sports injuries, degenerative injuries, and accidental injuries, this study aims to enhance the structural classification of football injuries. The study further updates the knowledge of injury processes and related prevention and treatment techniques for each category based on the most recent research findings. The objective is to provide a more coherent framework for understanding the fundamental reasoning and top preventive priorities related to football injuries. The goal of this work is to provide football practitioners with a better-organized perspective on injury typology while laying the theoretical groundwork for improving elite football performance and optimizing health management systems.

## 2 Literature search

This study adopted a narrative review approach rather than a systematic review. The primary aim was to provide a structured synthesis of injury types and prevention strategies among elite football players. A structured literature search was conducted across the following electronic databases: Google Scholar, PubMed, and SPORTDiscus (via EBSCOhost), covering the period from May 2000 to May 2025.

To ensure both breadth and sensitivity, the search strategy combined Boolean operators (AND, OR) and truncation symbols (*) for keyword combinations. Both Medical Subject Headings (MeSH) and free-text terms were used. Keywords included but were not limited to: “elite athletes” OR “professional footballers” OR “soccer” AND (“injury” OR “injuries” OR “injury epidemiology” OR “muscle” OR “tendon” OR “joint” OR “ligament” OR “muscle imbalance” OR “meniscus” OR “tendinopathy” OR “fracture” OR “concussion” OR “injury prevention” OR “injury intervention” OR “treatment” OR “rehabilitation training”). All keywords are used for searching in the title, abstract, and keyword fields.

The literature screening adhered rigorously to the established inclusion and exclusion criteria (refer to Table 1). Each publication underwent a review procedure comprising title, abstract, and full-text evaluations, and was included solely if it particularly featured elite football players and had usable statistics or pertinent intervention details. The reference lists of included research were meticulously examined to discover any other eligible studies that may have been overlooked in database searches.

**Table 1:** Screening process and inclusion and exclusion criteria.

Criteria Type	Description
Search the database	Google Scholar (Mainly); PubMed; SPORTDiscus (ancillary)
Search time range	May 2000 to May 2025
key words	“elite athletes” OR “professional footballers” OR “soccer” AND (“injury” OR “injuries” OR “injury epidemiology” OR “muscle” OR “tendon” OR “joint” OR “ligament” OR “muscle imbalance” OR “meniscus” OR “tendinopathy” OR “fracture” OR “concussion” OR “injury prevention” OR “injury intervention” OR “treatment” OR “rehabilitation training”).
Inclusion	(1) Studies involving elite or professional football players; (2) Content related to injury type, prevention, or rehabilitation; (3) Full-text available in English; (4) Original research (e.g., cohort, cross-sectional, RCT, Epidemiological research, Video Analysis Research).
Exclusion	(1) Non-elite populations (e.g., amateurs, adolescents); (2) Editorials, conference abstracts, reviews; (3) Animal/laboratory-only studies; (4) No relevant data on prevention or injury classification.

## 3 Occurrence and prevention strategies of general sports injuries in football

**Figure 1:**
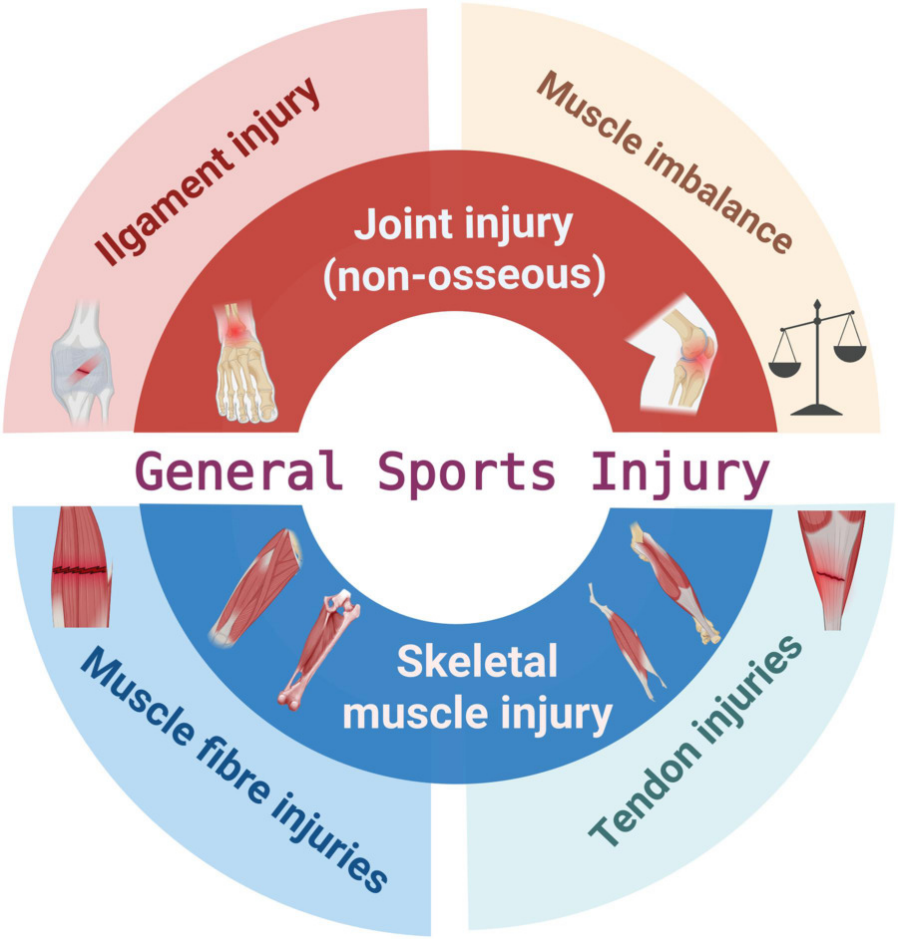
Schematic diagram of general sports injuries in football. The adductor icon in the panel is adapted from Corcoran et al. (148). Created using BioRender (ID: HA28P4FWQG).

Compared to training sessions, football games had a significantly higher frequency of injuries (23). Taking the 2022 World Cup as an example, the injury rate during the competition period was 20.6 per 1,000 h, while during training it was only 2.1 per 1,000 h (24). Moreover, elite football players are exposed to the combined demands of high-intensity training and frequent competition throughout the year, and this high exposure rate is associated with an increased risk of injury (25). The most common injuries in football involve the ankle and knee joints, as well as the muscles and ligaments of the thigh and lower leg (23). Mechanisms of injury and injury prevention measures vary because of the anatomical regions where injuries occur. Research indicates that exercise-induced muscle injuries are associated with repetitive high-intensity eccentric contractions, with prevention primarily focused on strength enhancement (26). However, joint (non-bone) injuries during athletic activity are often related to excessive joint displacement under high-load conditions (27), with prevention strategies typically involving flexibility and balance training (28). To provide a more comprehensive and accurate analysis of injury-related factors across different anatomical regions, including internal mechanisms and external situational triggers, and to guide the selection of more appropriate preventive approaches, the present study investigates preventable football injuries from two perspectives: skeletal muscle and joint-related injuries. Figure 1 presents a summary of the categories and sites of injuries sports injuries in football, predominantly concerning structural harm to skeletal muscles, joints, and tendons.

### 3.1 Injuries and prevention of general sports injuries involving skeletal muscles in football

The most frequently injured anatomical region in football is the lower limbs (23). According to statistics from the 2022 FIFA World Cup, muscle and tendon injuries were the most prevalent injury type, with hamstring strains being the most frequently diagnosed condition (24).

Skeletal muscle serves as the primary effector organ of the locomotor system, accounting for approximately 40% of total body weight and making significant contributions to various bodily functions (29). Its core function is converting chemical energy into mechanical energy to generate force and power, maintain body posture, and produce movements that affect activity (29). Skeletal muscle is naturally plastic, meaning that it can change both structurally and functionally in response to contractile activity (endurance exercise, electrical stimulation, denervation), loading conditions (resistance training, microgravity), substrate supply (nutritional interventions), or environmental factors (hypoxia) (30). These adaptive responses not only enhance muscle strength and flexibility but also optimize neuromuscular control and tissue repair capacity, ultimately contributing to a reduced risk of injury during athletic activity (30). Muscle fiber injuries and tendon injuries are the two categories into which skeletal muscle injuries in football are classified in this study based on the structural characteristics of muscle tissue. The underlying mechanisms of each type are examined independently, and related preventive measures are compiled and suggested.

#### 3.1.1 Muscle fiber injuries

Muscle fibers constitute the basic structural units of skeletal muscle, with multiple fibers forming muscle bundles that collectively compose skeletal muscle tissue (29). A muscle cell is represented by each muscle fiber, which is the fundamental cellular unit of a sarcomere (a muscle ganglion, the contractile apparatus necessary to generate force) (31).

Strains, partial tears, and muscle ruptures are among the most prevalent muscle fiber injuries in football (8). Most of these injuries occur during high-intensity activities such as sprinting, jumping, or striking (32). When the mechanical strain applied to the muscle exceeds its physiological capacity, internal stress within the muscle can increase by several orders of magnitude, leading to varying degrees of muscle fiber disruption, which is frequently accompanied by inflammation or damage to the surrounding connective tissues (33). Excessive eccentric loading is recognized as the most common cause of acute muscle fiber injuries (34). Table 2 delineates muscle fiber injuries frequently observed in elite soccer players throughout athletic endeavors, alongside the elevated prevalence of these injuries in particular sporting contexts and preventive strategies corroborated by extant literature. The hamstrings, adductors, quadriceps, and calf muscle groups are the most often injured muscle fibers (35), often linked to high-velocity running, abrupt directional changes, jumping, and explosive actions (36–39). Preventive therapies largely encompass eccentric training and injury prevention programs(e.g., FIFA1+) (40–43).

**Table 2:** Skeletal muscle injuries as general sports injuries in football: scenarios & training-based prevention strategies.

Site of Injury	Common muscle fiber injuries				Common tendon injuries	
	Hamstring	Adductor	Quadriceps	Calf muscles	Patellar tendon	Achilles tendon
Injury Scenario	Associated with sprinting, abrupt stops and turns, kicking, or high-speed hip flexion with knee extension	Change of direction and kicking (Mainly side-foot kicking)	Sprint running, forceful shooting, and long-range passing	Acceleration, high-intensity running, and jumping movements	Rapid change of direction, vertical jumping	Sustained running, acceleration sprints, and vertical jumping
Preventive Measures	Eccentric hamstring strengthening (Nordic Hamstring Exercise), Injury Prevention Programs (FIFA11+)	Eccentric adductors strengthening (Copenhagen adduction exercise)	Eccentric quadriceps strengthening (Eccentric Squat/Eccentric Step-Downset), Injury Prevention Programs (FIFA11+)	Calf muscle explosive strength training (Squat Jumps/Weighted Calf Raises), Balance training	Quadriceps-Hamstrings Eccentric Training (Eccentric Walking Lunge/Eccentric Bulgarian Split Squat), Patellar Tendon-Protective Balance Training (Squat on Balance Board）	Calf muscle explosive strength training (Weighted Squat Calf Raises/Eccentric Single-Leg Calf Raise), Achilles Tendon-Protective Balance Training (single-leg balance/Single-Leg Romanian Deadlift）
Reference	Arnason et al. (14) Askling et al. (44) Gronwald et al. (36) Daneshjoo et al. (40)	Hrysomallis et al. (41) Jensen et al. (42) Serner et al. (38)	Santos et al. (37) Valera-Garrido et al. (43) Daneshjoo et al. (40)	Green et al. (39) Owen et al. (145)	Murtagh et al. (51) Kraemer et al. (15) Larsson et al. (53) Jonsson et al. (54)	Kraemer et al. (15) Villa et al. (48) Hoenig et al. (52)

The injury scenario for Achilles tendon injuries is deduced from biomechanical concepts and clinical experience, due to the absence of direct empirical investigation.

Previous studies have demonstrated that implementing eccentric strengthening exercises during the preseason can optimize physical capacity and increase muscle strength, thereby reducing the incidence of muscle injuries during competition. Askling et al. (2003), for instance, reported that the incidence of hamstring-related injuries in athletes was substantially reduced by preseason eccentric strengthening of the hamstrings (44). In a similar vein, Ishøi et al. (2016) discovered that the Copenhagen Adduction exercise in preseason training programs increased hip adductor strength and reduced the likelihood of adductor injuries during matches (45).

#### 3.1.2 Tendon injuries

Tendons, mostly consisting of highly organized collagen fibers, are essential to the human musculoskeletal system, facilitating force transmission and governing the dynamic interplay between muscles and bones, while also offering mechanical protection (46).

In football, common tendon injuries include tendon rupture and tendon avulsion (8), most frequently affecting the patellar tendon (47) and the Achilles tendon (48). These injuries are typically associated with single-leg support and jumping actions (47, 48). Tendon ruptures (avulsions) during football activities typically arise from acute overload, wherein the applied force surpasses the tendon's ultimate tensile strength, leading to fiber failure and rupture (14). Reviewing the mechanisms of tendon overuse injury induction in animal models, it is mainly due to excessive mechanical loading (49, 50). Table 2 summarizes the prevalent tendon injuries sustained by elite football players during competition, identifies the high-risk sports situations linked to these injuries, and outlines the exercise training preventive methods endorsed by available literature. The patellar tendon and Achilles tendon are especially susceptible to damage, frequently occurring in high-impact, repetitive load situations such as running, jumping, and directional shifts (51, 52). Associated preventive strategies encompass eccentric resistance training and balance training, designed to augment tendon load-bearing capacity and enhance coordination of lower limb muscles (15, 53, 54).

Prior studies have shown that eccentric strength training, whether conducted independently or alongside balance training, can significantly reduce the incidence of tendon injuries. Kraemer et al. (2009) discovered that integrating proprioceptive balance training into football programs markedly reduced the occurrence of tendon injuries in athletes (15). Likewise, Langberg et al. (2007) demonstrated that high-load eccentric exercise could alleviate degenerative alterations in tendons (55).

### 3.2 Injuries and prevention of general sports injuries involving joint (non-bone) in football

Joints, comprising the articular capsule and articular cartilage, serve as the points of articulation between bones and are essential components of the human musculoskeletal system (56). Joints enable flexible movement between bones while ensuring stability and support during motion (57). In football, the lower limbs are particularly engaged due to the need for frequent sprinting (with and without the ball), directional changes, jumping, and kicking (shooting and passing). Consequently, joint injuries in football primarily affect the knee and ankle, often arising from mechanical stress or impacts in these areas (28).

The knee joint is a complex hinge structure made up of the femur, tibia, and patella, allowing for flexion-extension in the sagittal plane and varus-valgus rotation in the frontal plane (58). Common football-related knee injuries include ligamentous injuries and cartilage damage (59).

The ankle joint consists of the articulating surfaces of the distal tibia and fibula with the talar trochlea, facilitating coordinated rearfoot motion (60). Rearfoot motion typically occurs across three primary planes: sagittal (plantarflexion–dorsiflexion), frontal (inversion–eversion), and transverse (internal–external rotation) (61). Due to the frequent ball-contact actions in football and the relatively weak structure of the lateral ankle ligaments (e.g., the anterior talofibular ligament), inversion forces can frequently lead to sprains (62). In the 2022 FIFA World Cup, ankle sprains accounted for 17% of all documented injuries (63).

Joint stability is primarily provided by ligaments, while the surrounding musculature plays a secondary, yet supportive, role. Together, they ensure reliable joint function (58). This study will examine prevalent joint injury mechanisms, occurrence scenarios, and effective preventative techniques in football, emphasizing two essential stabilizers of joint integrity: the periarticular muscles and ligaments.

#### 3.2.1 Impaired joint mobility caused by ligamentous injuries

Ligaments are pieces of dense connective tissue that are dominated by collagen fibers that give them a high tensile strength (64). Their principal role is to inhibit excessive or aberrant joint motions, thus preserving joint stability (64). In football, the most common joint(non-bone) injuries are ligament injuries associated with sprains (10). During the 2022 FIFA World Cup, ligament injuries accounted for 13% of all reported injuries, predominantly occurring in the knee and ankle joints (10).

The knee joint relies on the ACL to prevent anterior displacement of the tibia relative to the femur, while the posterior cruciate ligament restricts posterior displacement (59). Consequently, ACL tears constitute one of the most serious knee injuries in football (65). Medial collateral ligament (MCL) injuries are also frequent, while lateral collateral ligament and posterior cruciate ligament injuries are relatively rare (63).

Ankle injuries in football frequently occur due to player-to-player contact, particularly from a direct impact to the medial side of the lower leg or ankle (66). Lateral ankle sprains are the most common form of ankle injury, with the anterior talofibular ligament typically being the first structure to be compromised (67).

Most ligament injuries in football occur when muscles fail to adequately absorb ground reaction forces. Sustained high-impact forces over a short period may increase joint loading and result in ligament rupture (61). Rarer ligament injuries generally result from substantial external impacts during intense match situations. Table 3 delineates ligament injuries frequently observed in elite football players within the context of general sports injuries, including high-risk sports scenarios and preventive exercise training approaches substantiated by available literature. Ligament injuries (ACL and MCL), commonly arise from abrupt halts, swift directional shifts, or landing maneuvers, especially during offensive and defensive engagements and sliding tackles (66, 68, 69). Associated preventive strategies encompass muscle balance training and neuromuscular training, designed to augment ligament load-bearing capacity and enhance joint stability (70–74).

**Table 3:** Joint (non-bone) injuries as general sports injuries in football: scenarios & training-based prevention.

Site of Injury	Joint dysfunction resulting from ligament injury			Muscle imbalance-induced joint injury	
	Knee joint		Ankle joint (anterior talofibular ligament)	Knee joint	Ankle joint
	Anterior cruciate ligament	Medial collateral ligament			
Injury Scenario	Sudden deceleration and directional changes, pressing/tackling, jump-landing manoeuvres	pressing/tackling and being tackled (Three-quarters of MCL injuries occur due to contact mechanisms)	Player-to-player contact: tackling and being tackled, Jump-landing	pressing/tackling and being tackled	pressing/tackling and being tackled; Faulty landing technique post-jump
Preventive Measures	Neuromuscular training, Quadriceps-Hamstring Strengthening Training, Warm-up training plan(Core-PAC)	Balance training (single-leg stance/multidirectional single-leg jumps), Neuromuscular & Proprioceptive Training, coordination training (agility ladder drills)	Neuromuscular training program, Proprioceptive balance training (Single-Leg Calf Raise on Board/Ball Toss on Balance Board), FIFA 11+	Progressive and periodized Integrated Neuromuscular Training (INT) program, Flexibility development training (High Knees/Butt Kicks), Dynamic Stretching, Individualized Resistance Strength Programs	Proprioceptive balance training (wobble board training), Strength training (hopping exercises), Flexibility development training（Ankle Circles）
Reference	Fischer et al. (70) Francesco et al. (68) Celebrini et al. (71)	Buckthorpe et al. (69) Lundblad et al. (72)	Andersen et al. (66) Verhagen et al. (73) Wentao et al. (74)	Croisier et al. (85) Costa et al. (86) Śliwowski et al. (88) Veeck et al. (89)	Andersen et al. (66) Mohammadi Nia Samakosh et al. (146)

Core-PAC stands for Core Position and Control movement strategy. The preventive strategies listed are proposed based on the injury mechanisms and situational patterns identified in video analysis studies (e.g., Buckthorpe et al., 2021), rather than on direct interventional evidence for MCL injury in professional football players.

Previous studies have demonstrated that strength and balance training can significantly reduce the incidence of ligament injuries. Myklebust et al. (2003) demonstrated that possible to prevent anterior cruciate ligament injuries with specific neuromuscular training (75). Fischer et al. (2006) demonstrated that proprioceptive balancing board interventions effectively prevented repeated ankle sprains (70). In addition, Waldén, M. et al. (2012) demonstrated that implementing neuromuscular warm-up protocols significantly decreased the incidence of ACL injuries among adolescent female football players (76).

#### 3.2.2 Joint injuries caused by muscle imbalances

Joint sprains are prevalent injuries in football and have garnered significant attention due to their elevated recurrence rates (77). Previous studies indicate that muscle strength and balance play key roles in preventing targeted muscular injuries (78), while muscle imbalance can increase the incidence of joint injuries in athletes (79). Therefore, it can be suggested that muscle imbalance affects joint stability and thereby increases the incidence of joint (non-bone) injuries. Imbalance in muscle strength typically denotes an abnormal bilateral asymmetry between homologous muscle groups and a disruption in the agonist-antagonist ratio (78).

The quadriceps cover the entire front of the thigh, extending from the hip to the knee joint (80), and function as the primary muscles for knee extension (58). The hamstrings, situated on the posterior aspect of the thigh, extend from the ischial tuberosity to the lower leg and function as the principal muscles for knee flexion. Additionally, the sartorius, gluteus maximus, gracilis, and gastrocnemius also assist in knee flexion and extension (81). Previous studies have shown that the hamstring-to-quadriceps (H/Q) strength ratio is a significant risk factor for hamstring strains in football (58) and is also strongly correlated with non-contact ACL injuries, particularly among female players (82).

The ankle dorsiflexor group (primarily tibialis anterior) is located on the front of the lower leg and is responsible for dorsiflexion of the ankle, while the plantar flexor group, mainly situated on the back of the lower leg, facilitates plantar flexion (83). The tibialis anterior and posterior muscles facilitate ankle inversion, whereas the peroneus longus and brevis muscles are responsible for eversion movements (83). Similarly, in ankle injury studies, Fousekis K et al. (2012) identified that eccentric isokinetic strength asymmetry between dorsiflexion and plantar flexion is an independent risk factor for non-contact ankle sprains in football players (84).

Regarding injury prevention, Prior studies prove that muscle imbalance-related injuries can be mitigated through compensatory strategies or training modifications (85), and that Dynamic Stretching can enhance joint stability and reduce the occurrence of joint injuries during play (86).

Interestingly, aside from muscle imbalances, Witvrouw E et al. (2003) concluded that increased tightness raises the risk of subsequent injury in certain muscle groups (hamstrings, quadriceps), even though flexibility independently influences other muscle injuries (adductors, calves) (87). Table 3 delineates the muscle imbalances that precipitate joint injuries in elite soccer players, contextualized within general sports injuries, and recommends preventive training measures grounded in existing evidence. Effective prevention and control strategies must prioritize comprehensive training programs that encompass lower limb muscle group strength coordination, balance, and neuromuscular control to improve joint stability and motor control capabilities (85, 86, 88, 89).

In summary, numerous studies have investigated and validated preventive strategies for general sports injuries, including muscle fiber injuries, tendon injuries, joint and ligament injuries, and injuries caused by muscle imbalances. However, the levels of evidence and strengths of recommendation for these interventions vary considerably. To provide sports medicine clinicians, coaches, and athletic trainers with a clearer understanding of the scientific basis and practical value of different preventive measures, this study summarizes and classifies the levels of evidence and grades of recommendation for these strategies according to the Oxford Centre for Evidence-Based Medicine (OCEBM) system (Table 4).

**Table 4:** Levels of evidence and grades of recommendation for prevention strategies of general sports injuries in elite football.

Injury type	Preventive strategy	Level of Evidence (OCEBM)	Grade of Recommendation	Key references
Hamstring Injuries	Nordic Hamstring Exercise	I	A	Arnason et al. (14) Askling et al., 2003; (44)
	Injury Prevention Programs (FIFA11+)	II	A	Daneshjoo A. et al., 2013; (40)
Adductor Injuries	Copenhagen Adduction Exercise	II	A	Jensen et al. (42)
Quadriceps Injuries	Eccentric quadriceps strengthening	II	B	Valera-Garrido et al. (43)
	Injury Prevention Programs (FIFA11+)	II	B	Daneshjoo et al. (40)
Calf Muscle Injuries	Injury Prevention Programs	II	B	Owen et al. (145)
Patellar Tendon Injuries	Quadriceps-Hamstrings Eccentric Training	I	A	Larsson et al. (53) Jonsson, P. et al., 2005; (54)
	Balance Training	II	B	Kraemer R. et al., 2009; (15)
Achilles Tendon Injuries	Balance Training	II	B	Kraemer R. et al., 2009; (15)
ACL Injuries	Neuromuscular training	I	A	Fischer et al., 2006; (70) Grimm et al. (17)
MCL Injuries	Injury Prevention Programs (FIFA11+)	V	C	Makuch et al. (147)
Anterior Talofibular Ligament Injuries	Proprioceptive balance training	III	B	Verhagen et al. (73) M. Wentao et al., 2023; (74)
Muscle Imbalance	Strength training	II	B	Śliwowski et al. (88) Mohammadi Nia Samakosh et al. (146)
	Proprioceptive balance training	III	B	Costa et al. (86)

## 4 Treatment and rehabilitation of degenerative injuries in football

Elite athletes often begin specializing in a sport at an early age, pursuing intensive training in a single sport for more than eight months per year (90). However, early specialization exposes many athletes to an increased risk of overuse injuries (91). These chronic degenerative changes resulting from prolonged high-load training are characteristic of overuse injuries, which differ fundamentally in pathophysiology from acute injuries and are often difficult to prevent through conventional training interventions (92). Research on football injuries has revealed that, alongside those that can be prevented by training, a range of joint-related degenerative injuries exists, including meniscus lesions, osteochondral lesions of the talus (OLT), tendinopathy, and bursitis (8).

It should be noted that the same anatomical structure may sustain either degenerative or traumatic injuries. For example, meniscal injuries in elite athletes can be either degenerative, resulting from cumulative loading, or traumatic, caused by acute contact (9). The ESSKA consensus emphasizes the importance of distinguishing these two mechanisms due to their different etiologies and treatment strategies (93). While degenerative lesions are generally managed conservatively, acute traumatic tears—though classified as contact injuries—often require surgical intervention and structured rehabilitation, and are therefore frequently considered alongside degenerative injuries in clinical practice (94).

**Figure 2:**
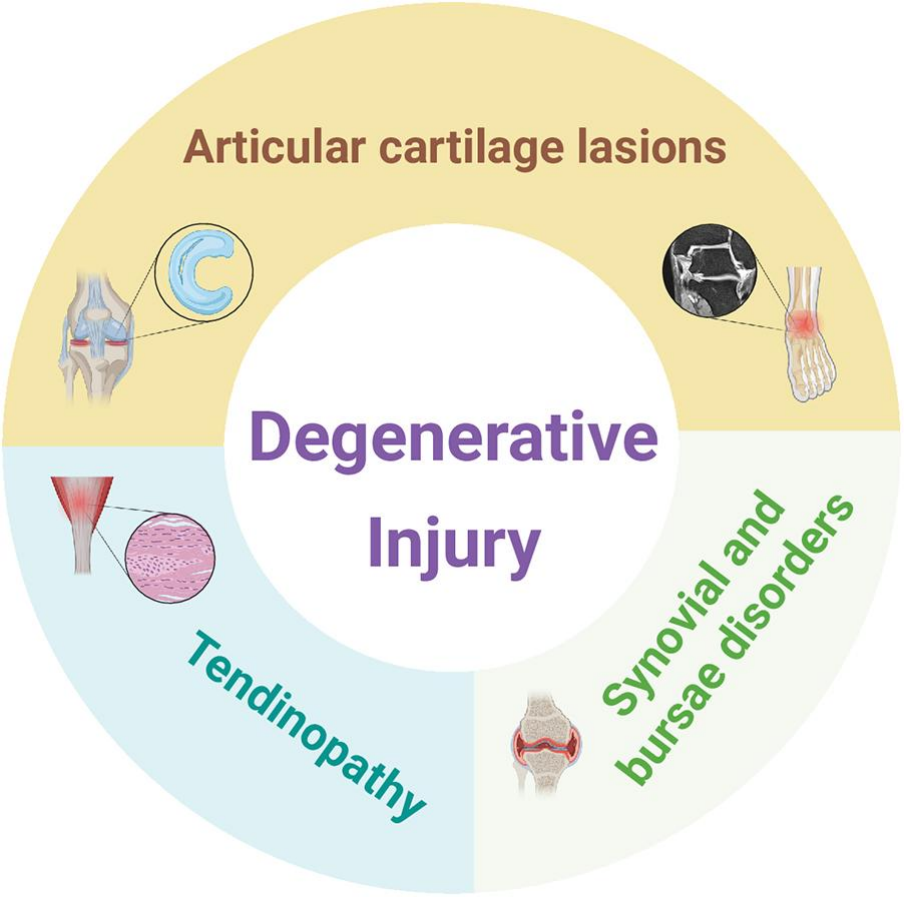
Classification diagram of degenerative injury in football. The magnetic resonance tomography of the ankle joint icon in the panel is adapted from Biehl et al. (149). Created using BioRender (ID: BX28L8JGDC).

In addition to meniscal injuries, Figure 2 further illustrates the common degenerative injuries observed in football players, which are primarily concentrated in the lower limb joints and encompass different categories of injury. Degenerative injuries often require prolonged drug treatment or surgical intervention, and even after treatment, rehabilitation training is still necessary to return to the field. Severe degenerative injuries can even affect lifelong health and career longevity. This study will summarize and analyze the treatment methods and phased rehabilitation procedures for prevalent degenerative injuries in football, offering theoretical references for athletes' health management.

### 4.1 Meniscus injuries and OLT in football

Meniscal injuries are the most common pathological knee injuries (95). Between 1996 and 2006, approximately 850,000 meniscal operations were performed each year in the United States, accounting for 10% to 20% of all orthopaedic procedures, with an incidence rate of 61 cases per 100,000 individuals (95). Chomiak J. et al. (2000) examined severe injuries in elite football players in the Czech Republic and determined that meniscal injuries constituted 8% of serious injuries, rendering it one of the most common degenerative injuries (18).

The mechanism of meniscal injury typically involves varus or valgus forces acting directly on a flexed knee when the foot is in contact with the ground, often accompanied by internal rotation of the femur (19, 96). Specifically, a valgus force applied to a flexed knee can result in medial meniscus tears (96). Treatment strategies should be customized to the severity and location of the meniscal tears.

For degenerative meniscal tears, most guidelines recommend at least 3–6 months of non-operative management (anti-inflammatory and analgesic medications, quadriceps strengthening, and unloader bracing) (97, 98). Acute traumatic meniscal tears often require operative treatment (meniscectomy, meniscal repair, meniscal reconstruction, meniscal scaffolds) depending on the injury characteristics (98). For athletes to re-establish their pre-injury performance levels and resume playing, it is essential to undergo high-quality rehabilitation following surgery.

**Figure 3:**
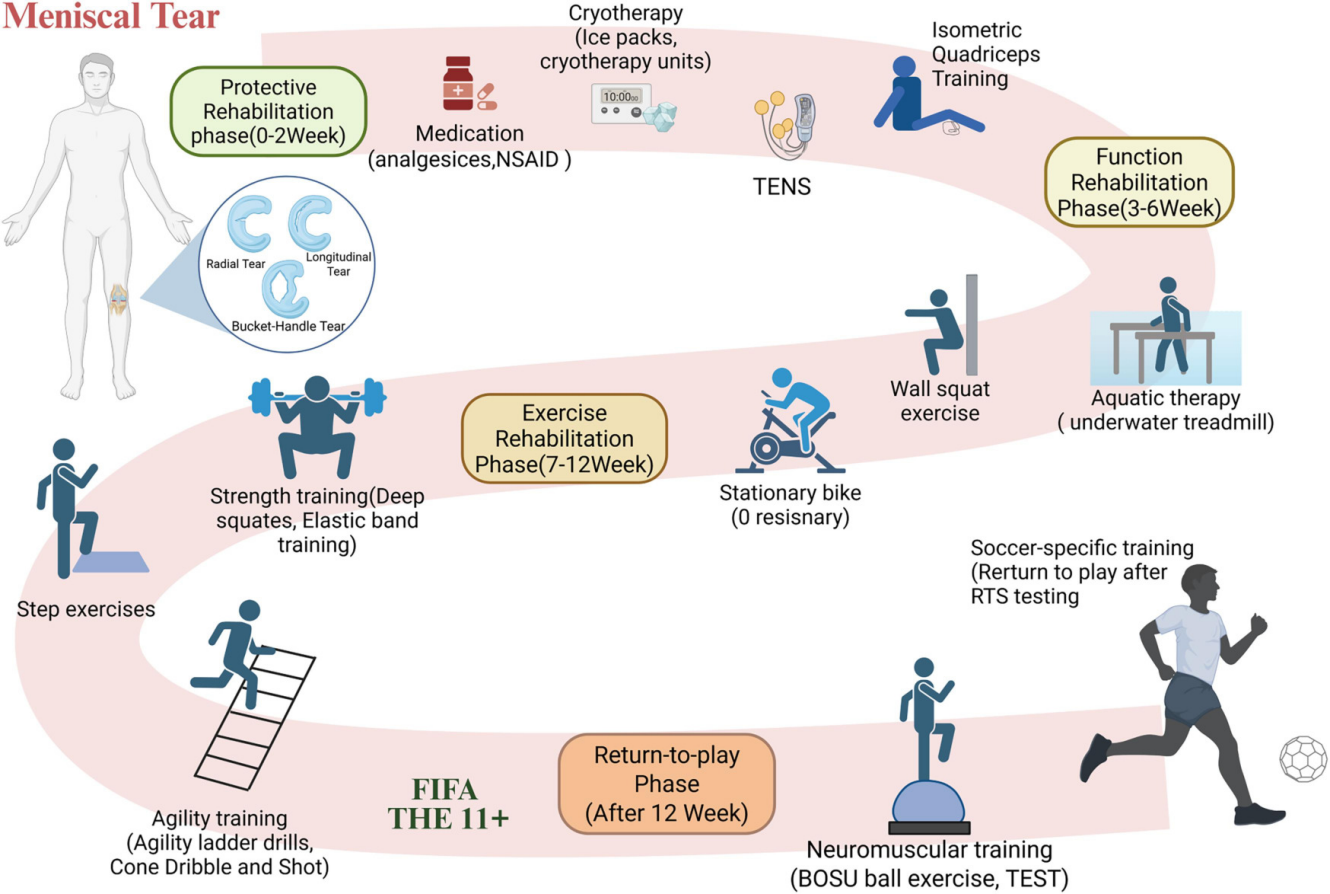
Flowchart of the phased rehabilitation protocol after meniscal tear surgery. This figure outlines the phase-based rehabilitation protocol following meniscal tear surgery, including protective, functional, exercise, and return-to-play phases, incorporating. TENS, aquatic therapy, strength and agility training, and football-specific interventions. NSAID: Non-steroidal anti-inflammatory medications. TENS: Transcutaneous electrical nerve stimulation. Isometric Quadriceps training: performed with a towel placed under the knee; the thigh is contracted and pressed downward. TEST: Task-explicit sensorimotor training. RTS testing: return-to-sport testing. FIFA 11+: FIFA 11 + warm-up training program. Created using BioRender (ID: HH28D5WDPS).

According to current recommendations, post-operative rehabilitation after meniscal surgery is divided into three key phases. During the protective rehabilitation phase (0–2 weeks), the focus is on quadriceps activation, crutch weaning, and achieving a range of motion between 0° and 30°. In the functional rehabilitation phase (3–6 weeks), the objective is to enhance lower extremity strength, improve balance and proprioception, and range of motion up to 90°. The exercise rehabilitation phase (7–12 weeks), emphasizes the gradual increase in training burden to regain athletic ability. Additionally, football-specific training should be integrated to standardize movements that are specific to the sport (97, 99–102). Figure 3 depicts a comprehensive rehabilitation training regimen for meniscus tears post-surgery, with distinct rehabilitation techniques chosen based on the specific objectives of each phase.

In addition to meniscus injuries, common cartilage lesions in football players also include OLT (62). Although the anatomical locations differ, both meniscal tears and OLT share similar etiologies, involving either chronic mechanical overload or acute trauma (103). OLT accounts for 50% to 70% of all acute ankle sprains and fractures and may present mechanical symptoms such as ankle clicking or joint locking (104). For asymptomatic or mildly symptomatic OLT cases, nonoperative treatment is typically recommended, including rest, ice application, temporary offloading, and bracing for joint malalignment (104). For traumatic osteochondral lesions (e.g., OLT) patients, operative treatment is required (marrow stimulation, osteochondral autograft, and osteochondral allograft) (103).

**Figure 4:**
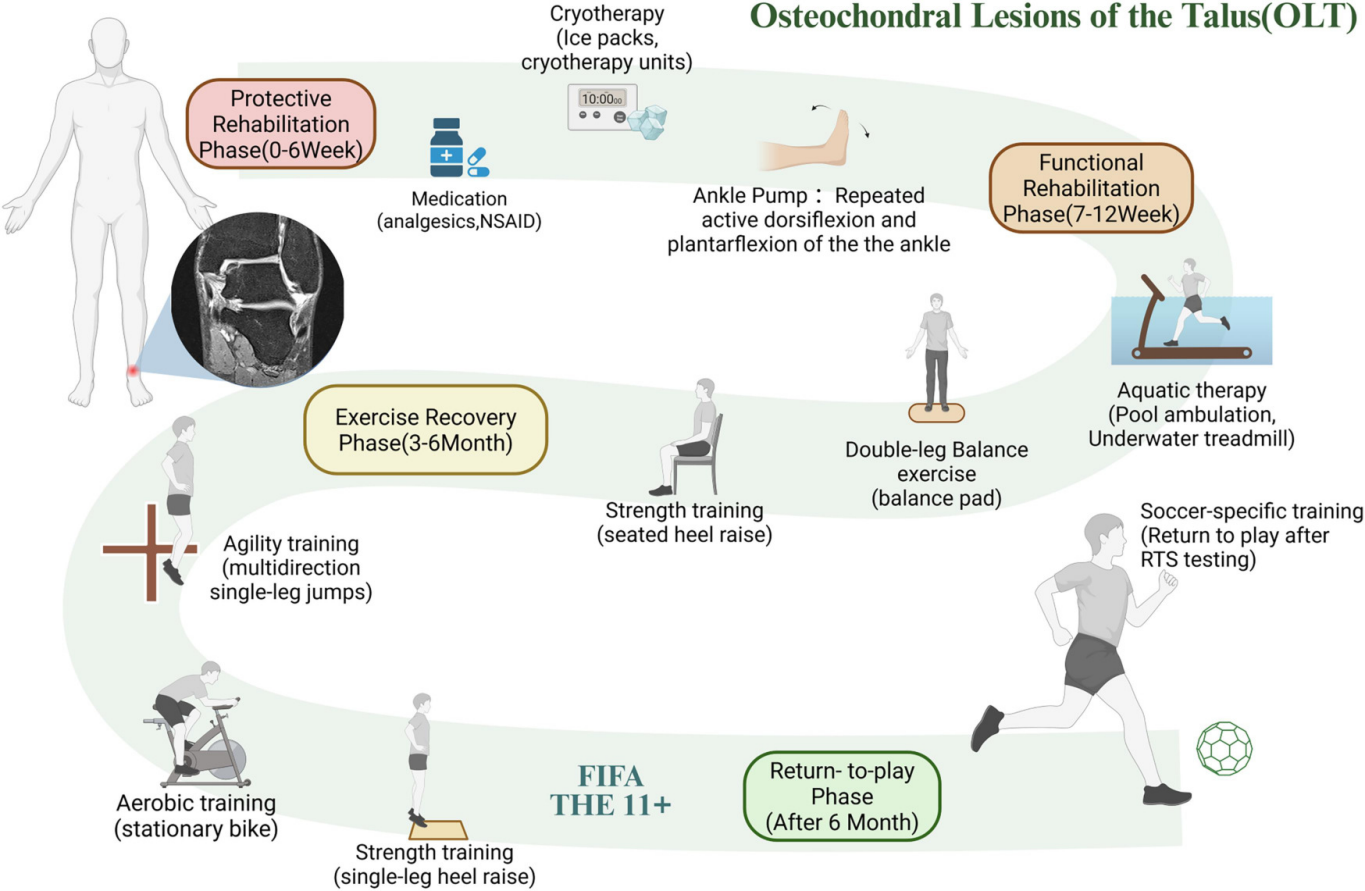
Flowchart of the phased rehabilitation protocol after OLT surgery. This figure presents the staged rehabilitation for talar OLT, including protective, functional, exercise recovery, and return-to-play phases, involving ankle pump, balance, aquatic training, and sport-specific assessment. RTS testing return-to-sport testing. The magnetic resonance tomography of the ankle joint icon in the panel is adapted from Biehl et al. (149). Created using BioRender (ID: NS28D5W7JL).

Post-operative OLT rehabilitation is also staged, similar to meniscal injury rehabilitation. However, the protective phase is extended to six weeks due to the ankle's increased load-bearing demands. Additionally, proprioceptive training is heavier. In the protective rehabilitation phase (0–6 weeks), the objective is to enhance lower extremity strength, emphasize balance training, and restore full range of motion. During the functional rehabilitation phase (7–12 weeks), underwater treadmill training can be employed to restore range of motion and enhance lower limb strength. In the exercise rehabilitation phase (months 3–6), training should be gradually intensified, focusing on proprioceptive training and regaining athleticism. Football-specific exercises should be developed gradually, and football-specific movements must be standardized. Finally, during the return-to-play phase (after 6 months), gradually increase the intensity of football-specific training and complete the return-to-sport testing to return to play (105–107). Figure 4 illustrates an example of a rehabilitation training plan following OLT injury surgery, with specific rehabilitation methods selected for different rehabilitation goals.

It is important to emphasize that the intensity of rehabilitation training should be progressively increased in accordance with the individual's recovery conditions. Conversely, it may result in an elevated risk of re-injury, reduced recuperation effects, and an extended recovery period (9).

### 4.2 Tendinopathy in football

Tendons are subjected to significant mechanical tension and loads during sports exercise (108). The prevalence of tendinopathy has consistently increased as elite athletes continue to raise the intensity, frequency, and duration of their training to achieve higher performance (108). In football, the lower limb tendons endure prolonged periods of elevated load due to sudden stops, changes in direction, and kicking movements (23). Consequently, Achilles tendinopathy and patellar tendinopathy are common issues among professional players. Aiyegbusi A I et al. (2021) discovered that the prevalence of Achilles tendinopathy in Nigerian football players was as high as 15.9% (109). According to Hägglund M. et al. (2011), patellar tendinopathy accounted for 1.5% of all injuries among elite European football players between 2001 and 2009, with a notably high recurrence rate (110).

Tendinopathy is a clinical syndrome that usually, but not always, implies an overuse tendon injury and is characterized by pain, diffuse or localized swelling, and impaired function (110). The pathomechanism that is most widely accepted posits that protracted mechanical stress during athletic activity results in the degradation of extracellular matrix and the disruption of collagen fibers (111). When the rate of degeneration exceeds that of repair, it results in pathological tendon thickening or hardening (111, 112). Tendinopathies are frequently accompanied by inflammatory pathologies in the adjacent connective tissues (tendonitis, peritendinitis, and tenosynovitis) (113).

The initial course of action for tendinopathy is conservative treatment, with operative options being evaluated if nonoperative measures are unsuccessful (113). Treatment methods include:

(1) Eccentric training. In terms of therapeutic efficacy, Stanish W D et al. (1986), 200 patients performed daily eccentric loading exercises, with 44% reporting complete pain relief and 43% reporting significant improvement (114). Mechanistically, eccentric training enhances the abundance of cytoskeletal proteins within tendon cells, thereby improving biomechanical properties during healing (115). It also appears to reduce glycolytic metabolism in fibrotic tendons, potentially linked to improved peripheral vascularization (116).
(2) Nonoperative Treatment: oral non-steroidal anti-inflammatory medications (NSAIDs), extracorporeal shockwave treatment/low-intensity pulsed ultrasound, and corticosteroid injections, etc (112–114, 117).
(3) Operative Treatment: endoscopic procedures, and minimally invasive tendon stripping/tenotomies, etc (117).

### 4.3 Synovial and bursae disorders in football

Bursitis is a common cause of musculoskeletal pain, with prevalent types including prepatellar, olecranon, trochanteric, and retrocalcaneal (118). Such injuries often occur in high-intensity sports. It has been estimated that synovial/bursae injuries (Synovitis/capsulitis) accounted for 4% of all injuries at the 2022 FIFA World Cup in Qatar (24). These injuries were more common in players who had been training in shooting for an extended period, which is linked to iliopsoas bursa friction (119). The onset of bursitis is directly related to repeated friction from adjacent muscles or tendons; when mechanical loading exceeds the tissue's tolerance threshold, it triggers an inflammatory response (118). The risk of bursitis more than triples in athletic populations who engage in persistent high-load activities (120).

Treatment should follow a “conservative-first, stepwise-escalation” approach. Nonoperative treatment, including cryotherapy, activity modification, and NSAIDs, is usually the first line of intervention (118). Surgical options, such as bursectomy, may be considered if symptoms persist, as in cases of refractory trochanteric bursitis (118). Individualized rehabilitation programs should be incorporated as part of post-treatment care to reduce the likelihood of recurrence.

## 5 Contextualization and prevention and control of accidental injuries in football

Due to the high physical demands and intense body contact inherent in football matches (121), most players often engage in risky and aggressive behaviors (heading duels and tackling) despite being aware of the potential dangers (122). These accidental injuries are typically classified as contact injuries, caused directly by physical collisions or external forces. Nevertheless, the professional football culture of “playing through injury” may hinder players' ability to accurately assess the risks associated with injuries (123), thereby increasing the probability of accidental injuries, including fractures and head and neck trauma. Data from the 2022 FIFA World Cup revealed that the incidence of accidental injuries (bone fractures, skin lacerations, and head/neck injuries) was 0.7 per 1000 h, which corresponds to approximately 12% of all injury cases (24). Moreover, studies have found that, compared to amateur football players, most contact injuries occur among professional football players (50%–70%) (124). Within this category, aside from head and neck injuries and fractures, soft tissue contact injuries (such as contusions, strains, and sprains) are recognized as a major category of concern (124, 125). Although the incidence of accidental injuries is relatively low, they often result in prolonged absences from training or competition (24). To reduce the incidence of accidental injuries, the following measures are recommended:

(1) Strengthen safety education for athletes to raise awareness of high-risk movements and enhance personal safety and injury prevention knowledge.
(2) Optimize match regulations (126), and ensure that referees penalize dangerous play strictly and promptly (127).
(3) Emphasize the importance of protective equipment (shin guards, goalkeeper gloves, and protective headgear).
(4) Evidence suggests that the prevention of soft tissue contact injuries should go beyond general measures for accidental injuries and include structured training programs focusing on dynamic stability and controlled body contact, which enhance neuromuscular control and joint stability in collision scenarios and thereby reduce the incidence of contact-related muscle and ligament injuries (128, 129).

This research analyses the causes of unintentional injuries, particularly those arising from contact mechanisms, to offer preventive recommendations for the safety of athletes.

### 5.1 Accidental head and neck injuries in football

In elite football, heading is a crucial skill predominantly used for tackling and shooting (130). Heading is the second most effective shooting technique, accounting for 18.4% of all goals (131). Previous research has demonstrated that players execute between 1 and 9 headers per match (132). Between 1998 and 2004, concussions, neck strains, and facial contusions were the most common types of head and neck injuries in FIFA tournaments, with an incidence rate of 12.5 per 1,000 h of play (133). Head and neck injuries predominantly result from contact mechanisms, most commonly head-to-head collisions (38%) and elbow-to-head impacts (16%) (134).

It is particularly noteworthy that in a study of head and neck injuries in Qatar's professional football league over eight seasons, concussions accounted for approximately 30% to 45% of all head and neck injuries (135). Sport-related concussion, caused by external forces to the head or body, is a transient brain dysfunction typically classified as a mild traumatic brain injury (136). The accumulation of multiple mild injuries may lead to long-term neurological disorders, including chronic traumatic encephalopathy, cognitive dysfunction, and depression (136). Consequently, they are a priority for prevention and control.

The following preventive measures are also implemented to reduce the potential danger to brain health from headers in football. (1) Neck training can be implemented to enhance head stability, thereby decreasing the likelihood of injury Gillies L. et al. (2022) demonstrated that a progressive neck strengthening program for athletes reduced head and neck injuries, including concussions (137). (2) The burden on the cranium may be alleviated through the development and utilization of protective gear, such as protective headgear.

### 5.2 Accidental fractures in football

Elite football players experienced a fracture incidence of 0.27 per 1000 h (95% CI 0.25 to 0.30) from 2001 to 2013 (138). The average professional football team may experience 1 to 2 fractures per season (138). The wrist was the most common site of fractures in the upper extremities, with a prevalence of 60%. Conversely, lower extremity fractures were more likely to necessitate hospitalization, despite accounting for only 32% of cases (139). In the occurrence of upper extremity fractures in football, goalkeepers have a 7-fold higher incidence compared to non-goalkeeper players, with an average extension of more than 1 week of out-of-play time (140). For common fractures like distal radius fractures, patients have a recovery rate of 79%; however, the average time to return to play is 9 weeks (141). The tibia and fibula are the primary bones involved in lower limb fractures in football, with 61% of affected players experiencing fractures to both bones. It's important to note that transverse or short oblique fractures account for 95% of tibial fractures, which are most frequently the result of direct impact during tackling/tackled (142). Although 80% of players with tibial fractures eventually accomplish full recovery, the rehabilitation period can last up to 38 weeks, and only 73% of them return to their pre-injury level of performance (141).

Fracture prevention strategies in football focus on two main approaches. (1) Emphasizing the importance of protective gear: shin guards are essential for preventing tibial shaft fractures, and goalkeeper gloves help protect against hand fractures (143). Experimental evidence by Tatar Y. et al. (2014) showed that shin guards can absorb up to 95% of impact force and reduce impact strain by up to 51% (143). (2) Prioritizing natural grass over artificial turf for matches. According to Calloway S. P. et al. 2004), players are at a higher risk of foot injuries on artificial turf compared to natural grass surfaces (144).

## 6 Limitations of the current research

Although this review provides a structured perspective on common injury types and intervention strategies in football, several limitations remain.

(1) Due to the large number of keywords and alternative terms involved in this study, and the fact that most references were retrieved through Google Scholar—which lacks a built-in electronic search engine—it was not feasible to provide a detailed literature screening process or a full PRISMA flow diagram.
(2) Due to substantial heterogeneity among the included studies in terms of study populations, intervention methods, and outcome measures, a meta-analysis could not be conducted. As a result, this review is limited to descriptive synthesis, making it difficult to provide clear quantitative conclusions.
(3) The scope of this review is relatively broad, covering multiple types of structural injuries; however, it does not provide detailed stratification by injury type, phase, or severity. As a result, some recommendations are inevitably generalized and should be interpreted with caution in practical applications.
(4) The proposed categorization of injuries into three types—general sports injuries, degenerative injuries, and accidental injuries—entails some degree of overlap. For instance, chronic tendinopathy may exhibit both pathological and training-related characteristics. Further interdisciplinary consensus is needed to refine and standardize these classifications.
(5) The lack of distinction between contact and non-contact mechanisms may limit the applicability of targeted prevention strategies, as exemplified by ACL injuries.
(6) This study did not stratify injuries by temporal stage (acute, sub-acute, or chronic) or by severity, although these factors play a critical role in shaping treatment strategies and guiding the course of rehabilitation.
(7) This study did not comprehensively differentiate the traumatic and degenerative etiologies and mechanisms of all structural injuries, but instead discussed representative cases such as meniscal lesions. This approach may reduce the precision and practical utility of the conclusions for injury prevention and clinical management.
(8) The evidence base for the prevention and treatment recommendations in this study is limited, with most evidence derived from small-sample observational studies or expert opinion, and lacking support from high-quality randomized controlled trials. Therefore, the robustness and generalizability of the conclusions remain somewhat limited.

## 7 Conclusions

The high incidence of sports injuries among elite football players underscores the urgent need to establish comprehensive prevention and treatment strategies. However, inconsistencies in the current injury classification frameworks have hindered effective clinical and performance-based decision-making. This review proposes a revised classification, dividing injuries into three main categories: general sports injuries, degenerative injuries, and accidental injuries. Each type involves distinct mechanisms and challenges, necessitating targeted intervention approaches.

General sports injuries—such as hamstring strains, ACL tears, and ankle sprains—can be effectively prevented through eccentric strengthening, neuromuscular control training, and balance exercises. Degenerative injuries, including meniscal lesions, OLT of the talus, and tendinopathies, often require pharmacological or surgical treatment, followed by individualized, phase-based rehabilitation programs. Although accidental injuries such as concussions and fractures are inherently unpredictable, their risk can be mitigated using protective equipment, safety education, and rule modifications.

By introducing this adjusted classification system, practitioners, coaches, and sports medicine professionals can better identify injury risks early, design individualized injury prevention programs, and implement targeted rehabilitation strategies aligned with football-specific biomechanical demands. Future research should focus on evidence-based, position-specific, and sex-specific injury prevention strategies to enhance player safety, prolong peak performance, and support sustainable career development in elite football.
